# Antimony Selenide Solar Cells Fabricated by Hybrid Reactive Magnetron Sputtering

**DOI:** 10.3390/nano13152257

**Published:** 2023-08-05

**Authors:** Daniel Brito, Pedro Anacleto, Ana Pérez-Rodríguez, José Fonseca, Pedro Santos, Marina Alves, Alessandro Cavalli, Deepanjan Sharma, Marcel S. Claro, Nicoleta Nicoara, Sascha Sadewasser

**Affiliations:** 1 International Iberian Nanotechnology Laboratory, Av. Mestre José Veiga, 4715-330 Braga, Portugal; daniel.brito@inl.int (D.B.); pedro.anacleto@inl.int (P.A.); perez.rodriguez.ana@usal.es (A.P.-R.); jose.fonseca@inl.int (J.F.); pedro.h.2111@hotmail.com (P.S.); marina.alves@inl.int (M.A.); a.cavalli.phd@gmail.com (A.C.); deepanjan.sharma@infinitesima.com (D.S.); marcel.santos@usc.es (M.S.C.); nicoleta.nicoara@inl.int (N.N.); 2 Departamento de Física e Astronomia, Faculdade de Ciências, Universidade do Porto, Rua do Campo Alegre s/n, 4169-007 Porto, Portugal; 3 Nanotechnology Group, Department of Fundamental Physics, University of Salamanca, 37008 Salamanca, Spain; 4 Centre of Physics of Minho and Porto Universities (CF-UM-UP), Azurém Campus, 4800-058 Guimarães, Portugal

**Keywords:** hybrid sputtering, Sb_2_Se_3_, solar cell, sputtering, thin-film photovoltaics

## Abstract

The fabrication of Sb_2_Se_3_ thin-film solar cells deposited by a pulsed hybrid reactive magnetron sputtering (PHRMS) was proposed and examined for different growth conditions. The influence of growth temperature and Se pulse period were studied in terms of morphology, crystal structure, and composition. The Sb_2_Se_3_ growth showed to be dependent on the growth temperature, with a larger crystal size for growth at 270 °C. By controlling the Se pulse period, the crystal structure and crystal size could be modified as a function of the supplied Se amount. The solar cell performance for Sb_2_Se_3_ absorbers deposited at various temperatures, Se pulse periods and thicknesses were assessed through current-voltage characteristics. A power conversion efficiency (PCE) of 3.7% was achieved for a Sb_2_Se_3_ solar cell with 900 nm thickness, Sb_2_Se_3_ deposited at 270 °C and Se pulses with 0.1 s duration and period of 0.5 s. Finally, annealing the complete solar cell at 100 °C led to a further improvement of the V_oc_, leading to a PCE of 3.8%, slightly higher than the best reported Sb_2_Se_3_ solar cell prepared by sputtering without post-selenization.

## 1. Introduction

To enable the wide-spread large-scale use of photovoltaics (PV) supporting a clean energy system, it is necessary to develop solar cells based on earth-abundant, non-critical and non-toxic elements and raw materials. Sb_2_Se_3_, consisting of the earth-abundant elements Sb and Se, is a binary compound with only one stable phase and excellent optoelectronic properties, such as 1.2 eV bandgap, high-absorption coefficient (>10^5^ cm^−1^), reasonable carrier mobility (~10 cm^2^ V^−1^ s^−1^) [1], and carrier lifetime (~60 ns). Sb_2_Se_3_ has attracted considerable attention as a promising material for thin-film solar cells due to these favorable optoelectronic properties.

**Figure 1:**
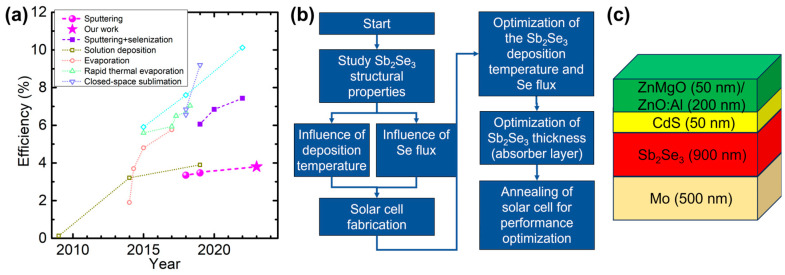
(**a**) Evolution of efficiency of Sb_2_Se_3_ solar cells prepared by different deposition technologies. Data from Refs. [1,3,4,5,6,7,8,9,10,11,12,13] and references therein. The lines are guides to the eye, connecting data points for the same deposition techniques. (**b**) Flowchart of the present study. (**c**) Schematic configuration of a complete Sb_2_Se_3_ solar cell structure with specific thicknesses.

However, despite the ideal theoretical efficiency of 32% [2], the best power conversion efficiency (PCE) achieved for Sb_2_Se_3_ solar cells is 10.12%, with a configuration similar to that of standard Cu(In,Ga)Se_2_ (CIGS) thin-film solar cells: Glass/Mo/MoSe_2_/Sb_2_Se_3_/CdS/ZnO/ZnO:Al [3]. The Sb_2_Se_3_ absorber layer was deposited by injection vapor deposition (IVD), leading to a highly [00l] oriented growth. The Sb_2_Se_3_ crystal orientation has a significant impact on solar cell performance. Chen et al. studied the basic properties of Sb_2_Se_3,_ concluding that the c-orientation presents higher electron mobility due to low scattering of charge carriers at grain boundaries [4], which is attributed to the one-dimensional crystal structure consisting in c-oriented ribbons connected by the van der Waals interaction. Consequently, grain boundaries separating columnar grains are free of dangling bonds, which minimizes recombination losses [5]. It has been shown that a MoSe_2_ seed layer can beneficially control Sb_2_Se_3_ orientation [6]. Sb_2_Se_3_ solar cells have been successfully deposited by a variety of techniques, such as closed-spaced sublimation [7] (CSS) with a previous record efficiency of 9.2%, rapid thermal evaporation [8] (RTE) with 7.04% efficiency by doping with Cu atoms to improve the carrier concentration, and by sputtering of a Sb layer followed by selenization [9] with a maximum of 7.43%. Sputtering presents several advantages, such as the possibility of fabricating a complete solar cell without breaking vacuum, good crystal quality, improved film uniformity, process reproducibility, and deposition over large area substrates. Large-area fabrication is crucial for the industrialization and production of solar cells. Most Sb_2_Se_3_ solar cells deposited by sputtering use Sb precursors followed by post-selenization [10,11,12]. Sputtering from a Sb_2_Se_3_ target results in a greater concentration of defects, which consequently require post-selenization. Rijal et al. concluded that post-selenization leads to higher PCE due to passivation of defects [13]. Furthermore, some theoretical studies have demonstrated that Se-rich synthesis could lead to a favorable V_oc_ [14], hence PCE. However, a good control over the Se supply during post-selenization would be required. Figure 1a shows the evolution of best efficiency values achieved for different deposition methods of the Sb_2_Se_3_ absorber layer.

Recently, a pulsed-hybrid reactive magnetron sputtering [15] (PHRMS) method was used for depositing CIGS solar cells. Here, Cu, In, and Ga were sputtered from a mixed target, while Se was evaporated from a valved cracker source in a pulsed manner. This method offers improved control over the Se amount owing to the use of a controllable valve.

This work presents the fabrication of Sb_2_Se_3_ solar cells in substrate configuration (Figure 1b,c) by a PHRMS process. The deposition process was optimized for substrate temperature, amount of Se supplied during deposition (by adjusting the Se pulses), and Sb_2_Se_3_ absorber layer thickness. An optimized solar cell with 900 nm absorber layer thickness deposited at 270 °C reached 3.67% power conversion efficiency.

## 2. Materials and Methods

### 2.1. Growth Procedure of Sb_2_Se_3_ Thin Films

Sb_2_Se_3_ thin films were deposited using a home-built hybrid sputtering-evaporation deposition system [15], consisting of three interconnected chambers for the deposition of (i) Mo, (ii) chalcogenide materials, and (iii) buffer and transparent conductive oxides (TCO). The Sb_2_Se_3_ layer was deposited in the chalcogenide chamber onto a soda-lime glass (SLG) substrate covered with a 500 nm Mo back contact. A Sb target (Testbourne) was sputtered, while Se was simultaneously evaporated in a pulsed manner (pulsed selenization). The temperature of the Se cell reservoir was 300 °C, and the cracker temperature was approximately 600 °C. Further information is provided in Refs. [15,16]. The Se valve opening was set to 9.0 mm and a magnetic actuator opens the valve, thus allowing the Se to enter into the chamber. In the present study, the Se pulse duration was fixed at 100 ms and the amount of Se was controlled by selecting the pulse period, with values from 1.2 s down to 0.5 s. The Sb sputtering was performed at a chamber pressure of 6.3 × 10^−3^ mbar, with a power of approximately 14.6 W, leading to a Sb growth rate of 14 nm/min. The deposition time was about 19 min, aiming for a Sb_2_Se_3_ thickness of 600 nm. The tested substrate temperature values ranged between 180–320 °C. These temperature values are nominal temperatures, measured by a thermocouple close to the back side of the substrate. The actual substrate temperature could be 50 °C higher, as suggested by an optical pyrometer for selected temperatures.

Two different sets were implemented to study the impact of the substrate temperature and the Se pressure by changing the pulses: Set A: substrate temperatures: 180, 225, 250, 270, and 320 °C, Se pulse period: 1.2 s; Set B: substrate temperature: 270 °C, Se pulse periods: 0.5 s, 0.8 s, and 1.2 s. 

### 2.2. Device Fabrication

A Mo back contact layer was deposited on soda-lime glass substrates (2.5 × 2.5 cm^2^) by dc-sputtering, consisting of a 100 nm adhesion layer deposited at high pressures (1.2 × 10^−2^ mbar) followed by a 400 nm layer deposited at 6.5 × 10^−3^ mbar, providing better electrical conductivity. Subsequently, the Sb_2_Se_3_ absorber layer was deposited, as described above, followed by a 50 nm thick CdS buffer layer by chemical bath deposition. Finally, the solar cell devices were completed with a transparent conductive oxide layer composed of 50 nm thick ZnO:Mg followed by a 200 nm thick ZnO:Al, both deposited by RF-magnetron sputtering at 5.6 × 10^−3^ mbar. A schematic illustration of the solar cell is shown in Figure 1c.

### 2.3. Characterization Techniques

The Sb_2_Se_3_ morphology and composition were studied by scanning electron microscopy (SEM, FEI Quanta 650 FEG, Hillsboro, OR, USA) equipped with energy-dispersive X-ray spectroscopy (EDS) for composition analysis. Crystal structure analysis was performed using X-ray diffraction (XRD, X’Pert PANalytical, Almelo, Netherlands). Additionally, confocal Raman spectroscopy (WITec alpha300 R, Ulm, Germany) was used to obtain information regarding the vibrational modes. The spectroscopy was implemented with a laser wavelength of 532 nm, power of approximately 3 mW and a 1800-line grating. The I-V characteristic curves for the solar cells were measured using a solar simulator (Oriel Sol3A class AAA, Newport, Irvine, CA, USA). The contact tips were directly placed on the ZnO:Al layer after dividing the 2.5 × 2.5 cm^2^ solar cell into approximately 20 individual solar cells with an average area of about 15 mm^2^.

## 3. Results and Discussion

### 3.1. Temperature Influence

**Figure 2:**
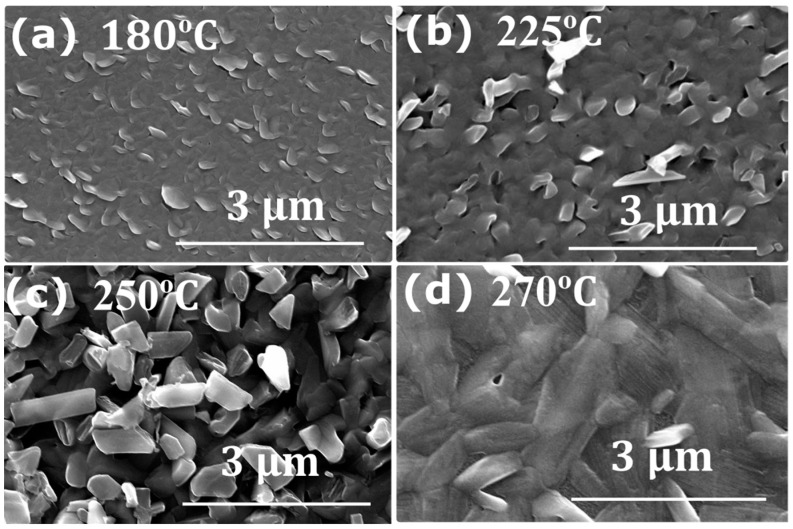
Top view SEM images of Sb_2_Se_3_ deposited by hybrid pulsed sputtering (Set A) at (**a**) 180 °C, (**b**) 225 °C, (**c**) 250 °C, and (**d**) 270 °C with Se pulse period of 1.2 s.

First, we studied the influence of the deposition temperature on the morphology of the Sb_2_Se_3_ thin films (sample Set A). The literature reports on optimal substrate temperatures in the range of 300–350 °C [17]. We used Sb_2_Se_3_ growth temperatures of nominally 180 °C, 225 °C, 250 °C, 270 °C and 320 °C in order to optimize the Sb_2_Se_3_ growth process at lower temperatures. Figure 2 shows top-view SEM images of the Sb_2_Se_3_ layer for all the samples. The lowest temperature of 180 °C leads to a mixed amorphous and crystalline Sb_2_Se_3_ layer. The partial crystallization at such low temperatures is attributed to the low melting point of Sb_2_Se_3_ [1]. Moreover, owing to its amorphous nature, the roughness of this layer is minimal. The crystal size, as observed in the SEM images, increases with higher substrate temperatures from ~200 nm at 180 °C to ~1 μm at 250 °C (Figure 2). The Sb_2_Se_3_ grown at 250 °C appears polycrystalline with preferential columnar growth, which we attribute to better nucleation with a preferential arrangement leading to better crystallinity and larger grain size. Further increasing the temperature to 270 °C leads to a uniform and continuous film, without pinholes or cracks, and with a larger crystal size of ~2 μm. For the highest growth temperature of 320 °C, no Sb_2_Se_3_ deposition was observed, which we attribute to the re-evaporation of Sb_2_Se_3_, due to its low-melting point. Table 1 presents the composition of the Sb_2_Se_3_ layer as analyzed by EDS. It is observed that the composition is not influenced by the growth temperature, in agreement with the single phase of Sb_2_Se_3_ composition.

**Figure 3:**
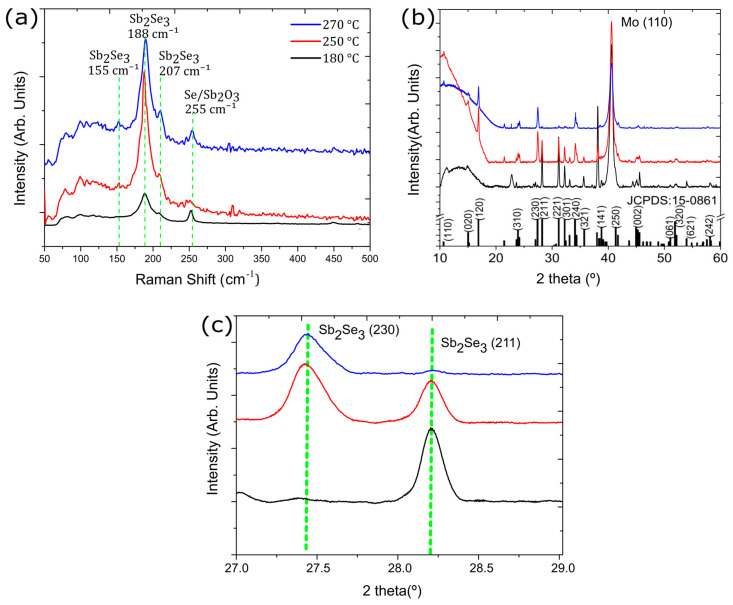
(**a**) Raman spectra of Sb_2_Se_3_ sample deposited at temperatures of 180, 250 and 270 °C. (**b**) XRD of Sb_2_Se_3_ sample deposited at temperatures of 180, 250 and 270 °C. (**c**) High-resolution of the Sb_2_Se_3_ planes (230) and (211). All samples were deposited with Se pulse period of 1.2 s.

To further study the materials structure, we performed Raman spectroscopy (Figure 3a) for the samples grown at 180 °C, 250 °C, and 270 °C. Three characteristic peaks were observed at 155 cm^−1^, 188 cm^−1^, and 207 cm^−1^, belonging to vibrational modes $B_{2g}^{1}$, $A_{g}^{3}$ and $B_{1g}^{2}$, respectively, in agreement with the literature [18]. We found that the intensity of the dominant peak for Sb_2_Se_3_, located at 188 cm^−1^, increases with temperature. At the lowest growth temperature of 180 °C, the signal presents a lower intensity, attributed to the quasi-amorphous layer of Sb_2_Se_3_, which is coherent with the SEM images. A small peak at 255 cm^−1^ was found to be an antimony oxide (α-Sb_2_O_3_) [18] or a Se-Se bond [19]. No other materials or contaminations were detected. The crystal structure of the Sb_2_Se_3_ layer was further studied by XRD (Figure 3b). The diffractograms indicate an orthorhombic Sb_2_Se_3_ crystal structure (space group Pnma), consistent with the literature [4,19,20,21]. All samples show a high-intensity peak at 40.5° related to the (110) plane of the Mo back contact. For the sample deposited at 180 °C, we detected a small peak at 22.7°, which is not in agreement with Sb_2_Se_3_, but could indicate metallic Sb [22]. At the relatively low temperature of 180 °C, the provided energy might be insufficient for the reaction of Sb and Se to Sb_2_Se_3_. No other material phases or contaminants were detected. Regarding the preferential growth orientation, there is a noticeable dependence on the temperature. At 180 °C, a preferential orientation can be seen along the (211) and (221) planes, which is known to be the most advantageous orientation for good solar cell performance due to the high-electron mobility along the c-axis or the [00l] direction [4]. For higher temperatures, the preferential orientation starts to change, favoring (120) and (230) planes (Figure 3c), which is not ideal for solar cells [17]. Planes parallel to the c-axis were not present in the sample at 180 °C. At 250 °C, it shows a mixture of both, confirmed by the SEM images (Figure 2c), which is considered a highly polycrystalline film. For a higher temperature of 270 °C, the (hkl) planes with *l* ≠ 0 are minimal.

### 3.2. Se Flux Influence

**Figure 4:**
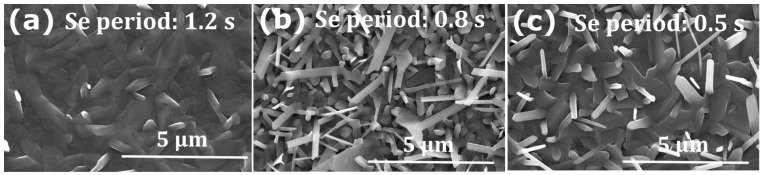
Top view SEM images of Sb_2_Se_3_ deposited at 270 °C by a hybrid pulsed sputtering with Se pulse periods of (**a**) 1.2 s, (**b**) 0.8 s, and (**c**) 0.5 s.

Several studies have shown that a post-selenization treatment improves the solar cell performance by filling detrimental Se vacancies and providing a better control of the crystallization [9,10,12,17,21,23]. In our PHRMS process, the amount of Se is controlled by the repetition rate of the 100 ms long Se pulses, i.e., by the pulse period. In order to supply a larger amount of Se to the growing Sb_2_Se_3_ layer, we studied the influence of shortening the pulse period, while maintaining the pulse duration (opening time of the Se valve) at 100 ms. Figure 4 shows SEM top-view images of the Sb_2_Se_3_ layer for different periods of the Se pulses, with a fixed growth temperature of 270 °C, (sample Set B). As observed previously, the Se pulse period of 1.2 s produces a smooth surface with a larger crystal size and a preferential growth parallel to the substrate [21]. With an increased amount of Se at a pulse period of 0.8 s, the crystal size of the Sb_2_Se_3_ film is reduced and the crystal shape significantly changed, showing narrow elongated grains. A further increase of the Se supply through a Se pulse period of 0.5 s leads to larger crystal size of ~2 µm, considerably larger than that reported in the literature [10,24]. The grains still exhibit an elongated shape.

**Figure 5:**
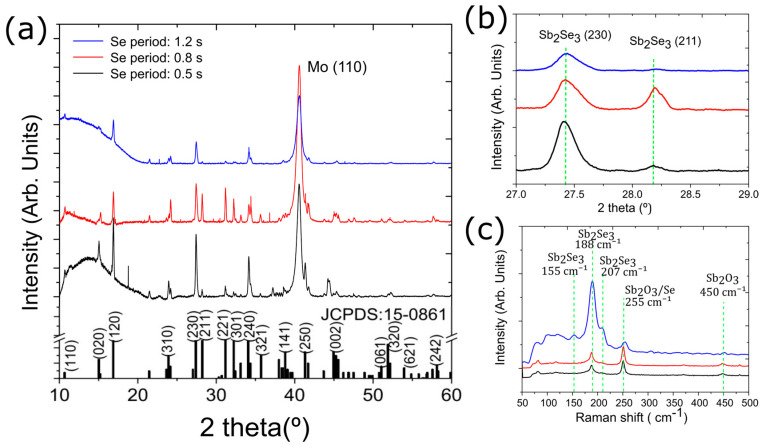
(**a**) XRD of Sb_2_Se_3_ samples deposited at 270 °C with pulse durations of 0.5 s, 0.8 s, and 1.2 s. (**b**) High-resolution XRD of the Sb_2_Se_3_ planes (230) and (211). (**c**) Raman spectra of Sb_2_Se_3_ samples. All samples were deposited at a temperature of 270 °C.

Figure 5 shows the XRD diffractograms and the Raman spectra for the Sb_2_Se_3_ samples from Set B. The XRD data in Figure 5a clearly show that the crystal orientation can be influenced by the amount of Se supplied during growth, in the present case, controlled by the pulse period. The Sb_2_Se_3_ layer was still highly polycrystalline with a mixture of (hkl) and (hk0) planes. The (211) plane peak, at 28.19°, and the (221) plane peak, at 31.16°, increased in intensity compared to the other planes. The sample with Se pulse period 0.5 s on the other hand, showed the opposite effect and exhibited a preferential growth orientation along the (120), (230), and (420) Sb_2_Se_3_ planes.

In Raman spectroscopy (Figure 5c), the samples produced with a higher Se supply showed a higher intensity of the 255 cm^−1^ peak, which is related to Sb_2_O_3_ [21].

### 3.3. Solar Cell Devices

To study the suitability of the prepared Sb_2_Se_3_ layers for solar cell applications, we fabricated solar cell devices by depositing a CdS buffer layer by chemical bath deposition and a window layer (ZnO:Mg and ZnO:Al) by RF sputtering, leading to the following device architecture: Mo/Sb_2_Se_3_/CdS/ZnO:Mg/ZnO:Al. Two sets of solar cells were fabricated, first, for a pulse period of 1.2 s, two growth temperatures were tested for the Sb_2_Se_3_ layer, 225 °C and 270 °C. Secondly, for a growth temperature of 270 °C, pulse periods from 0.5 to 1.2 s were tested.

**Figure 6:**
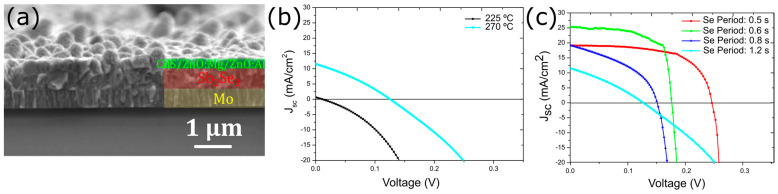
(**a**) SEM cross-section view of a complete Sb_2_Se_3_ solar cell, showing the Mo layer and Sb_2_Se_3_ layer covered by the window layer. (**b**) J-V curves for solar cells based on Sb_2_Se_3_ absorbers deposited at 225 °C and 270 °C (Se pulse period: 1.2 s). (**c**) J-V curves of devices with the Sb_2_Se_3_ absorbers deposited at 270 °C with various Se pulse periods.

A cross-sectional view of the solar cell fabricated from the Sb_2_Se_3_ layer deposited at 270 °C and with 0.8 s pulse period is shown in Figure 6a. The Sb_2_Se_3_ layer presents large vertical grains with a fairly rough surface, in agreement with the respective top-view SEM image (Figure 4b).

The J-V curves for the best-performing single cell of the studied deposition conditions are shown in Figure 6b,c. The solar cell with the Sb_2_Se_3_ layer deposited at 225 °C did not show any photocurrent, indicating a lack of carrier collection. Nevertheless, a diode behavior is clearly observed, confirming the formation of a p-n junction. Therefore, we attribute the lack of a photo current to a high concentration of defects (acting as recombination centers) due to the low-deposition temperature. Moreover, the small grain size is another possible reason for the low current, as it increases the number of grain boundaries and, consequently, the carrier recombination. Nevertheless, the solar cell prepared with the Sb_2_Se_3_ deposited at 270 °C does show a photocurrent and leads to a device efficiency of 0.45% (Table 2). Nevertheless, all device parameters, i.e., open-circuit voltage (V_oc_) of 0.126 V, short-circuit current density (J_sc_), and fill factor (FF) are far from values reported in the literature [10,13,25].

**Figure 7:**
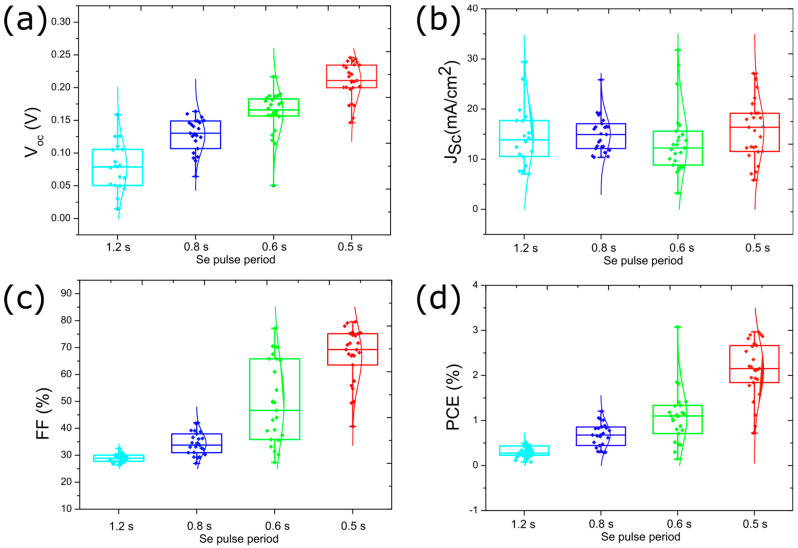
Box plots of the solar cell device parameters for different Se pulse durations during the deposition of the Sb_2_Se_3_ absorber layer at a temperature of 270 °C. (**a**) V_oc_, (**b**) J_sc_, (**c**) FF, and (**d**) PCE.

Likely, the formation of Se vacancies is responsible for the poor solar cell device performance observed for the cells with Sb_2_Se_3_ layers deposited with Se pulse period of 1.2 s. The J-V curves for the best-performing solar cell for a series of Se pulse periods are shown in Figure 6c with the device parameters provided in Table 2. The data shows that providing more Se during Sb_2_Se_3_ deposition leads to significantly better solar cell performance. The J-V curves show substantial improvement in all parameters, especially in the series and shunt resistances (Figure S1). A statistical representation (i.e., box plots) of all measured solar cells for the various Se pulse periods is shown in Figure 7.

The data shows a V_oc_ improvement with higher Se supply. The improvement in V_oc_ may be related to the difference in carrier concentration. Studies have shown that Se vacancies are promptly formed in Sb_2_Se_3_ [21]. The increased Se supply resulting from the shorter Se pulse periods is expected to reduce the formation of Se vacancies [19]. Nevertheless, even the best V_oc_ values (~0.21 V for the 0.5 s Se pulse period) are still relatively low compared to those reported for the best devices in the literature [3,6,13]. On the other hand, the J_sc_ does not vary considerably for the different Se pulse periods, with an average in the range of 13–17 mA/cm^2^. The fill factor (FF) is considerably improved from ~30% to ~70% using shorter pulse periods, which is associated with an improvement in the shunt resistance (Figure S1). Consequently, the solar cell average efficiency improved from less than 0.5% to 2.2%, with a maximum efficiency of ~3%. This efficiency value is comparable with that reported in the literature for the best Sb_2_Se_3_ solar cell deposited by sputtering (efficiency of 3.35%) [24].

### 3.4. Sb_2_Se_3_ Thickness Optimization for Solar Cell Performance

**Figure 8:**
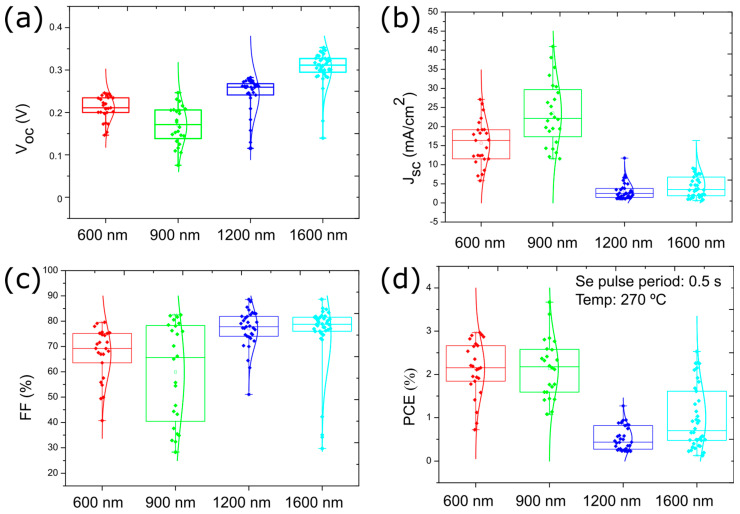
Box plots presenting the influence of the Sb_2_Se_3_ thickness on the solar cell device parameters (**a**) V_oc_, (**b**) J_sc_, (**c**) FF, and (**d**) power conversion efficiency. All solar cells were deposited at a temperature of 270 °C with Se pulse period of 0.5 s.

Further V_oc_ improvement might be obtained by optimizing the thickness of the Sb_2_Se_3_ absorber layer [23]. Therefore, a variation of the absorber layer thickness was carried out, maintaining the previously identified optimum deposition conditions of 270 °C with Se pulse duration of 0.5 s. Figure 8 shows the solar cell device parameters for four different thicknesses (600, 900, 1200, and 1500 nm). The J-V curve of the best-performing cell for each thickness is presented in Figure S2. The V_oc_ increases with thickness from 0.24 V to 0.31 V, which we attribute to the better light absorption and resulting increased charge carrier generation. The effect on FF was similar to that on V_oc,_ obtaining values closer to 80%. However, an excess thickness is harmful to the J_sc_. For Sb_2_Se_3_ layer thickness larger than 1 µm, the J_sc_ average dropped to below 5 mA/cm^2^. Even though the light absorption will be higher, the probability of charge-carrier recombination will also increase, the electron collection will be reduced, hence, reducing the J_sc_. The significant drop in J_sc_ leads to a drop in efficiency. Consequently, the higher J_sc_ and the lower V_oc_ for the sample with 900 nm Sb_2_Se_3_ thickness leads to similar efficiency values as the sample with a Sb_2_Se_3_ thickness of 600 nm. The series and shunt resistances are shown in Figure S3.

In summary, the ideal thickness would be between 600 and 900 nm, with a slightly improved efficiency of 3.67% for 900 nm Sb_2_Se_3_ thickness. Table 3 presents the solar cell parameters for the best performing solar cell for the tested Sb_2_Se_3_ thicknesses.

### 3.5. Solar Cell Annealing for Performance Improvement

Liang et al. [26] presented an annealing treatment after the formation of the Sb_2_Se_3_/CdS heterojunction, which was suggested to lead to a diffusion of Cd and S atoms into the absorber layer. It was observed that the V_oc_ increases, which the authors attributed to an increase of the doping concentration in the absorber layer. Cd atoms replace Sb atoms leading to p-type doping, and Se is partially replaced by S atoms, increasing the absorber layer’s band gap. 

Therefore, we studied the impact of an annealing treatment on our Sb_2_Se_3_ solar cells, annealing the complete solar cell devices for 15 min at temperatures of 100, 140, and 180 °C in air. This annealing series was carried out for the solar cells with the Sb_2_Se_3_ absorber layer deposited at 270 °C with Se pulse period of 0.5 s and for Sb_2_Se_3_ thicknesses of 600 and 900 nm.

**Figure 9:**
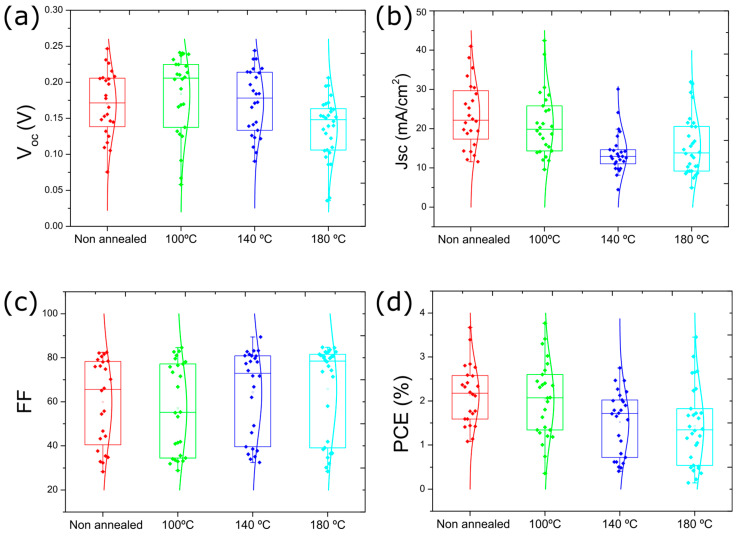
Box plots presenting the influence of annealing on the solar cell device parameters (**a**) V_oc_, (**b**) short-circuit current density (J_sc_), (**c**) fill factor (FF), and (**d**) power conversion efficiency (PCE) for Sb_2_Se_3_ solar cells with an absorber thickness of 900 nm deposited at 270 °C with Se pulse period of 0.5 s.

For the solar cells with 900 nm absorber thickness, the average V_oc_ of the solar cells improved upon annealing at 100 °C (Figure 9). For annealing at higher temperatures, V_oc_ starts to decrease. On the other hand, the annealing treatment deteriorates J_sc_ even for 100 °C. As a consequence, the PCE remained unchanged for annealing at 100 °C but decreases for annealing at higher temperatures. Nevertheless, a new efficiency maximum of 3.8% was achieved. The trends are similar for the solar cells with a Sb_2_Se_3_ thickness of 600 nm (Figure S4). This efficiency value (see star in Figure 1a) is higher than the previously achieved maximum efficiency of 3.47% [21] for sputter-deposited Sb_2_Se_3_ solar cells without a post-annealing step. The series and shunt resistances for the annealing study are shown in Figure S5.

## 4. Conclusions

In summary, the fabrication of Sb_2_Se_3_ solar cells by a PHRMS was demonstrated to be effective and efficient for the sputter deposition of Sb_2_Se_3_ solar cells. We confirmed that the morphology and the crystal orientation depend on deposition temperature and the Se supply during the growth of the Sb_2_Se_3_ layer. We conclude that larger Sb_2_Se_3_ grain sizes are obtained for growth temperatures higher than 250 °C.

With an optimized growth temperature of 270 °C, pulse period of 0.5 s (with Se pulse duration of 100 ms), and Sb_2_Se_3_ absorber layer thickness of 900 nm, a maximum efficiency of 3.67% was obtained, exceeding the previous maximum for sputter deposited Sb_2_Se_3_ solar cells. Finally, this value could be increased to 3.8% efficiency after annealing the complete solar cell at 100 °C. We expect that future work will lead to higher efficiency values for the single-step pulsed-hybrid reactive magnetron sputtering deposition process.

## Figures and Tables

**Table 1:** Atomic composition of the Sb_2_Se_3_ thin films and respective ratio Sb/Se measured by EDX.

Sample		Se (at. %)	Sb (at. %)	Ratio (Sb/Se)
Temperature (°C)	Se pulse Period (s)			
180	1.2	54.9	45.1	0.82
225	1.2	54.9	45.1	0.82
250	1.2	54.8	45.2	0.82
270	1.2	56.6	43.4	0.77
270	0.8	55.0	45.0	0.81
270	0.5	55.5	44.5	0.8

**Table 2:** Device parameters of the best solar cells with Sb_2_Se_3_ layers deposited at 270 °C with various Se pulse periods.

Sample (Best Cell)		Voc (V)	Jsc (mA/cm^2^)	FF (%)	PCE (%)
Temperature (°C)	Se Pulse Period (s)				
270	0.5	0.244	19.1	63.52	3.0
270	0.6	0.175	25.0	70.17	3.1
270	0.8	0.149	19.2	42.07	1.2
270	1.2	0.126	11.5	30.78	0.5

**Table 3:** Solar cell device parameters of the best solar cell with different Sb_2_Se_3_ absorber layer thicknesses. All the Sb_2_Se_3_ layers were deposited at 270 °C with Se pulse period of 0.5 s.

Absorber Layer Thickness (nm)	V_oc_ (V)	J_sc_ (mA/cm^2^)	FF (%)	PCE (%)
600	0.244	19.1	64	3.0
900	0.246	22.4	66	3.7
1200	0.241	7.3	72	1.3
1600	0.344	9.1	81	2.5

## Data Availability

The data presented in this study are available upon request from the corresponding author.

## Supplementary Materials

The following supporting information can be downloaded at: https://www.mdpi.com/article/10.3390/nano13152257/s1, Figure S1: Box plot presenting the influence of the different Se pulse evaporation on series resistance and shunt resistance of Sb_2_Se_3_ solar cells. All solar cells were deposited at a temperature of 270 °C; Figure S2: J-V curves of devices with the Sb_2_Se_3_ absorbers deposited at 270 °C with Se pulse period 0.5s for different Sb_2_Se_3_ thicknesses; Figure S3: Box plot presenting the influence of the Sb_2_Se_3_ thickness on series resistance and shunt resistance of Sb_2_Se_3_ solar cells. All solar cells were deposited at a temperature of 270 °C; Figure S4: Box plots presenting the influence of annealing on the solar cell device parameters (a) V_oc_, (b) short-circuit current density (J_sc_), (c) fill factor (FF), and (d) power conversion efficiency (PCE) for Sb_2_Se_3_ solar cells with an absorber thickness of 600 nm deposited at 270 °C with Se pulse period of 0.5 s; Figure S5: Box plot presenting the influence of the Sb_2_Se_3_ solar cell annealing on series resistance and shunt resistance of Sb2Se3 solar cells. All solar cells were deposited at a temperature of 270 °C.
