# Supplementary material for: Antimony Selenide Solar Cells Fabricated by Hybrid Reactive Magnetron Sputtering
Source: Nanomaterials (Basel). 2023 Aug 5;13(15):2257. doi: 10.3390/nano13152257 (PMC10420897; doi:10.3390/nano13152257)
Supplement: Supplementary file 1 [file nanomaterials-13-02257-s001.zip › nanomaterials-2505442-supplementary.pdf]

Supplementary Information

Antimony selenide solar cells fabricated by hybrid reactive magnetron sputtering

Daniel Brito[1,2], Pedro Anacleto[1], Ana Pérez-Rodriguez[1,3], Jose Fonseca[1], Pedro Santos[1], Marina Alves[1,4], Alessandro Cavalli[1], Deepanjan Sharma[1], Marcel Claro[1] Nicoleta Nicoara[1] and Sascha Sadewasser[1*]

[1] International Iberian Nanotechnology Laboratory, Av. Mestre Jose Veiga, Braga 4715-330, Portugal

[2] Departamento de Física e Astronomia, Faculdade de Ciências, Universidade do Porto, Rua do Campo Alegre s/n, 4169-007, Porto, Portugal

[3] Nanotechnology Group, Department of Fundamental Physics, University of Salamanca 37008, Salamanca, Spain

[4] Centre of Physics of Minho and Porto Universities (CF-UM-UP), Azurém Campus, Guimaraes ˜ 4800-058, Portugal

*corresponding author: Sascha.sadewasser@inl.int

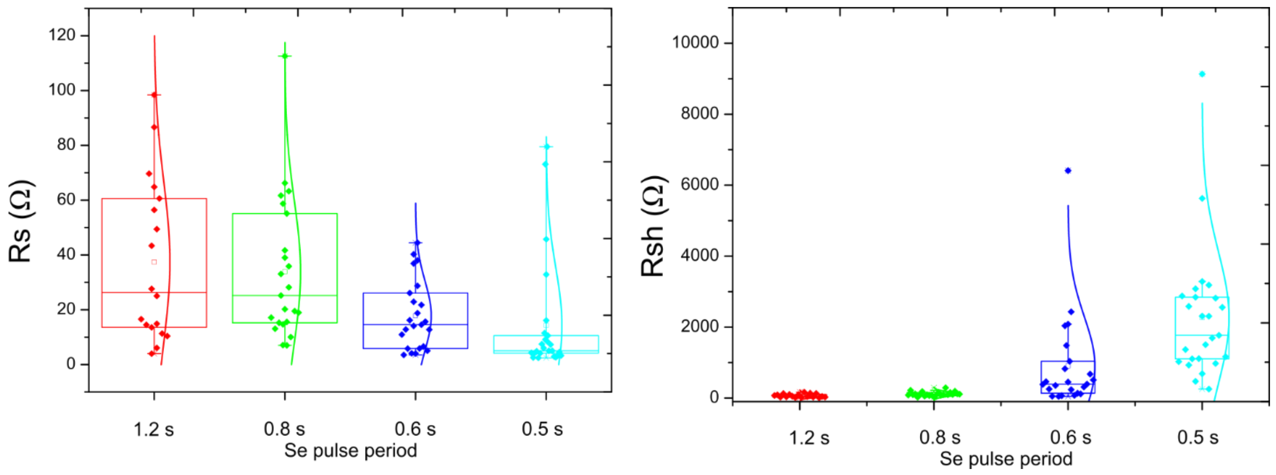

Figure S1: Box plot presenting the influence of the different Se pulse evaporation on series resistance and shunt resistance of Sb_2Se_3 solar cells. All solar cells were deposited at a temperature of 270 °C.

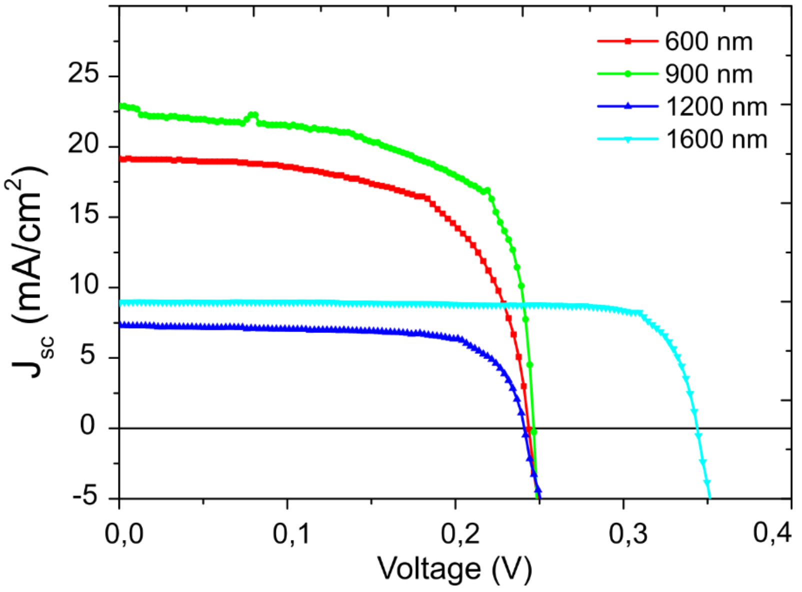

Figure S2: J-V curves of devices with the Sb_2Se_3 absorbers deposited at 270ºC with Se pulse period 0.5s for different Sb_2Se_3 thicknesses.

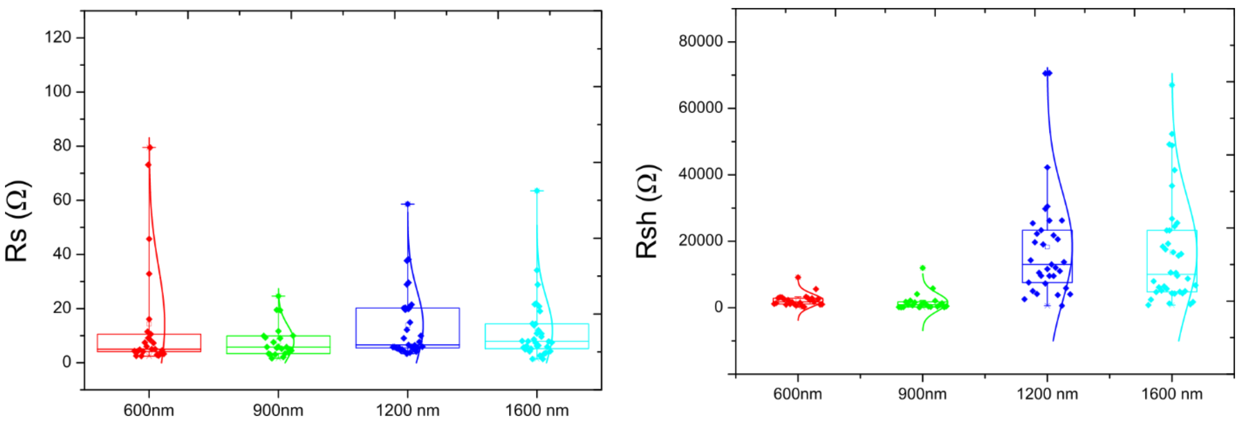

Figure S3: Box plot presenting the influence of the Sb_2Se_3 thickness on series resistance and shunt resistance of Sb_2Se_3 solar cells. All solar cells were deposited at a temperature of 270 °C.

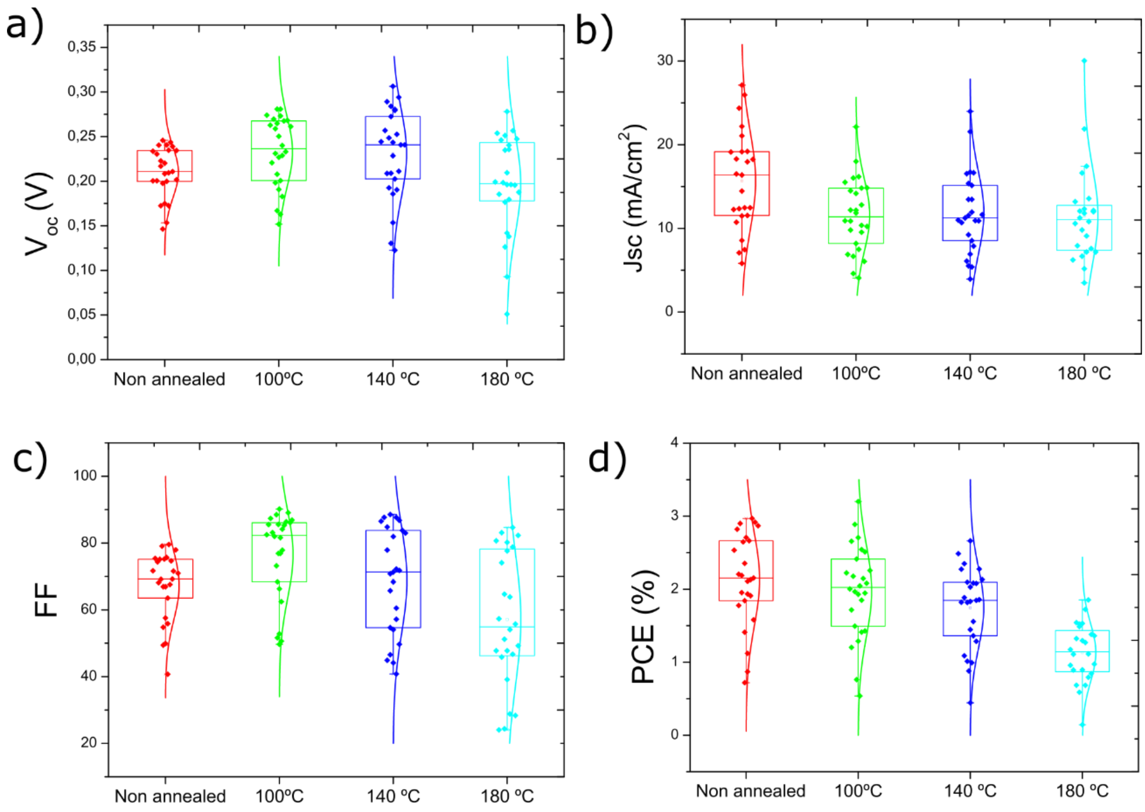

Figure S4: Box plots presenting the influence of annealing on the solar cell device parameters (a) V_{oc}, (b) short-circuit current density (J_{sc}), (c) fill factor (FF), and (d) power conversion efficiency (PCE) for Sb_2Se_3 solar cells with an absorber thickness of 600 nm deposited at 270 °C with Se pulse period of 0.5 s.

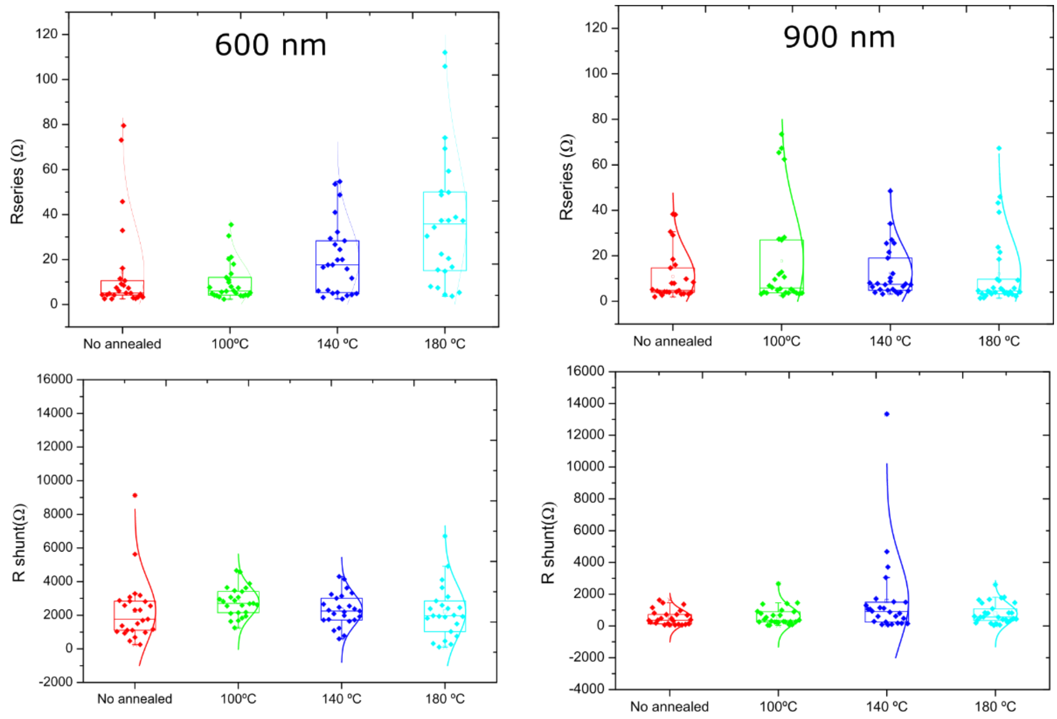

Figure S5: Box plot presenting the influence of the Sb_2Se_3 solar cell annealing on series resistance and shunt resistance of Sb_2Se_3 solar cells. All solar cells were deposited at a temperature of 270 °C.
